# Clinicopathologic features and surgical outcome of solid pseudopapillary tumor of the pancreas: analysis of 17 cases

**DOI:** 10.1186/1477-7819-11-38

**Published:** 2013-02-06

**Authors:** Xiao-Guang Wang, Quan-Fa Ni, Jian-Guo Fei, Zheng-Xiang Zhong, Peng-Fei Yu

**Affiliations:** 1 Department of Surgery, the Second Affiliated Hospital of JiaXing Medical College, JiaXing, 314000, China; 2 Department of Abdominal Surgery, Zhejiang Cancer Hospital, 38# Guangji Road, Zhejiang Province, Hangzhou, 310022, China

## Abstract

**Background:**

We summarize our experience of the diagnosis, surgical treatment, and prognosis of solid pseudopapillary tumors (SPTs).

**Methods:**

We carried out a retrospective study of clinical data from a series of 17 patients with SPT managed in two hospitals between October 2001 and November 2011.

**Results:**

All of the 17 patients were female and the average age at diagnosis was 26.6 years (range 11 years to 55 years). The tumor was located in the body or tail in ten patients, the head in five patients, and the neck in two patients. The median tumor size was 5.5 cm (range 2 cm to 10 cm). All 17 patients had curative resections, including seven distal pancreatectomies, five local resections, four pancreaticoduodenectomies, and one central pancreatectomy. Two patients required concomitant splenic vein resection due to local tumor invasion. All patients were alive and disease-free at a median follow-up of 48.2 months (range 2 to 90 months). There were no significant associations between clinicopathologic factors and malignant potential of SPT. Ki-67 was detected in three patients with pancreatic parenchyma invasion.

**Conclusions:**

The SPT is an infrequent tumor, typically affecting young women without notable symptoms. Surgical resection is justified even in the presence of local invasion or metastases, as patients demonstrate excellent long-term survival. Positive immunoreactivity for Ki-67 may predict the malignant potential of SPTs.

## Background

The solid pseudopapillary tumor (SPT) of the pancreas was first reported by Frantz in 1959 [1]. It is a rare neoplasm of low malignant potential, and accounts for approximately 1% of pancreatic tumors [2]. This tumor primarily affects young women and is usually treated with surgical resection [3]. After resection and follow-up, there is generally a relatively favorable prognosis. Recently, the number of cases reported in the literature has been steadily rising; however, the pathogenesis and guidelines for SPT treatment remain unclear. In this study, we report our clinical experience with 17 cases of SPTs.

## Methods

Between October 2001 and November 2011, 17 patients who underwent surgery for a pathologically confirmed SPT at the Department of Abdominal Surgery, Zhejiang Cancer Hospital and the Department of Surgery, Second Affiliated Hospital of JiaXing Medical College were reviewed retrospectively. Patients’ clinical presentation, radiological details, surgical data, pathological features, postoperative course, and long-term survival were collected and analyzed. Outpatient records combined with telephone interviews were used for follow-up.

Pathologically, SPT was defined as malignant if it demonstrated extrapancreatic invasion, distant metastases, pancreatic parenchymal invasion, or perineural or vascular invasion [4]. Univariate analyses of predictive features of malignancy were performed to compare clinicopathologic factors. All statistical analyses were performed with the computer program Statistical Package for Social Sciences (SPSS) 16.0 for Windows (Chicago, Illinois).

## Results

### Patient characteristics

All of the 17 patients were women, aged from 11 to 55 years (mean 26.6 years). The clinical presentation is unspecific, including abdominal pain (35.3%), abdominal discomfort (27.3%), abdominal distension (27.3%), back pain (11.8%), and vomiting (9.1%). Three patients whose SPT was found during routine physical examinations were asymptomatic. The patients had a median symptom duration of one month (range 5 days to 11 months). The tumors were 5.5 cm in diameter, on average (range 2 cm to 10 cm), and were located in the body or tail in ten patients, the head in five patients, and the neck in two patients. The clinical features of the 17 patients are listed in Table 1.

**Table 1:** Clinicopathologic features of 17 patients with SPTs

**Parameter**	**Patient number (*n *= 17)**	**%**
**Age** (years, mean (range))	26.6 (11-55)	
**Sex**		
Female	17	100%
Male	0	0%
**Symptoms**		
Abdominal pain	6	35.3%
Abdominal discomfort	4	27.3%
Abdominal distension	4	27.3%
Back pain	2	11.8%
Vomiting	1	5.9%
Asymptomatic	3	17.6%
**Size** (cm, mean (range))	**5.5 (2-10)**	
**Location**		
Body or tail	10	58.8%
Head	5	29.4%
Neck	2	11.8%
**Tumor feature**		
Solid and cystic	10	58.8%
Solid	7	41.2%
**Surgical treatment**		
Distal pancreatectomy	2	11.8%
Distal pancreatectomy + splenectomy	5	29.4%
Local resection	3	17.6%
Local resection + splenic vein	2	11.8%
**Resection**		
Whipple	4	23.5%
Central pancreatectomy	1	5.9%
**Follow-up** (months, mean (range))	48.2 (2-90)	
**Outcome**		
Alive	17	100%
Dead	0	0%

### Preoperative examination and diagnosis

**Figure 1:**
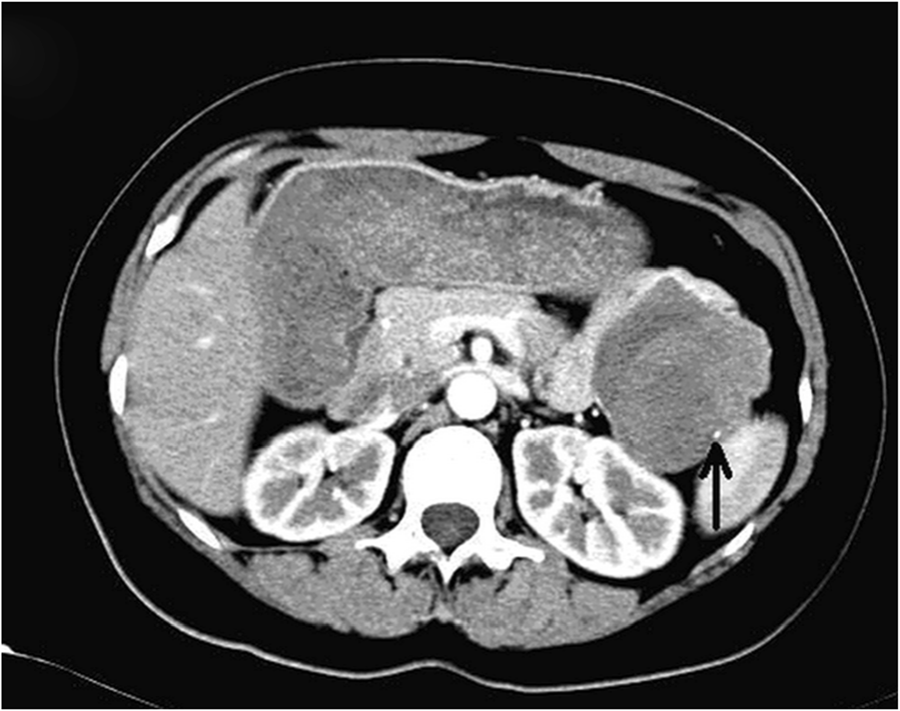
Contrast-enhanced CT shows a solid and cystic mass with a calcification (arrowed) located in the tail of the pancreas.

**Figure 2:**
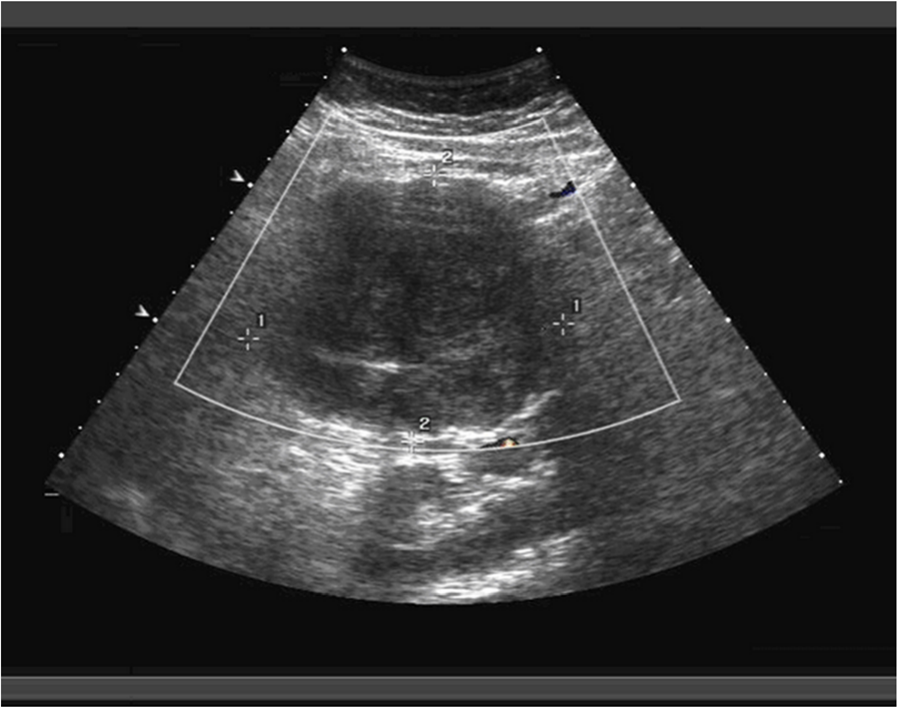
US of the left upper quadrant shows a large lesion located in the body and tail of the pancreas.

Radiological investigations were performed before operation, including computed tomography (CT) in twelve patients, ultrasonography (US) in eight patients, magnetic resonance imaging (MRI) in four patients, and US-guided fine needle aspiration cytology (FNAC) in two patients. Figures 1 and 2 show CT and US images of the SPT. The mass was described on cross-sectional imaging as heterogenous (solid and cystic) in ten patients and solid in seven patients. Calcifications were present in 4 of the 17 patients, while hemorrhage or necrosis was detected in 6 patients.

None of the patients had a definitive preoperative diagnosis and a correct diagnosis was made in six patients. Misdiagnoses included pancreatic adenocarcinoma (*n* = 6), cystadenomas (*n* = 3), islet cell tumors (*n* = 1), and pancreatic cyst (*n* = 1).

### Surgical data

All 17 patients underwent surgical exploration. Seven patients with lesions in the pancreas body or tail underwent a distal pancreatectomy, including two spleen-preserving resections. Five patients underwent local resection and two of them had concomitant splenic vein resection due to local tumor invasion. The remaining five patients underwent pancreaticoduodenectomy (Whipple, four cases) and central pancreatectomy (one case). Total surgery time ranged from 1.5 to 6.5 hours (mean 3.7 hours). Blood transfusion was needed in five patients during surgery; each patient received 2 units of blood.

All 17 patients had R0 resections and there were no surgical mortalities. Postsurgical complications occurred in five patients. One patient had pulmonary infection four days after surgery. Another patient had been found to have a pseudocyst in the first follow-up. Three patients had pancreatic leakage. The median postsurgical stay was 10.3 days (range 7 to 17 days).

### Pathological features

Grossly, the tumor is well encapsulated and is usually well demarcated from the pancreas. The cut surface shows large spongy areas of hemorrhage alternating with both solid and cystic degeneration. The tumors contain a mixture of solid, cystic, and pseudopapillary patterns in various proportions. Four patients had a malignant SPT; two patients had splenic vein infiltration and the other two had local invasion into the adjacent pancreatic parenchyma. No patients had lymph node metastasis.

**Figure 3:**
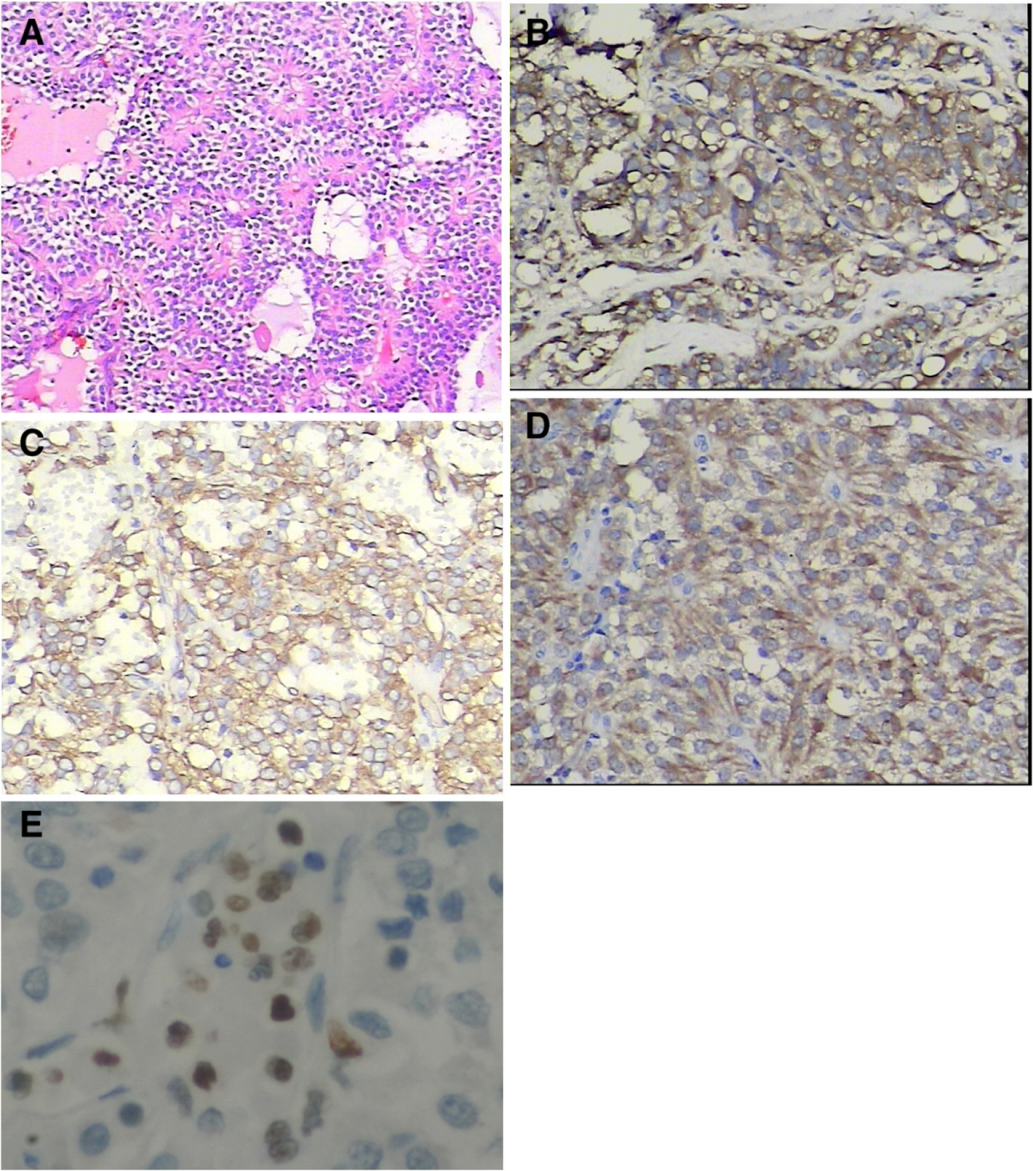
**Histopathology of SPTs.** (**A**) Sheets and cords of cells arranged around fibrovascular septa and pseudopapillary structures are formed. (H&E × 100). (**B**) Immunohistochemical staining for α1-antitrypsin (original magnification × 400). (**C**) Immunohistochemical staining for vimentin (original magnification × 400). (**D**) Immunohistochemical staining for neuron-specific enolase (original magnification × 400). (**E**) Immunohistochemical staining for Ki-67 (original magnification × 400).

Immunohistochemical studies were performed in all 17 cases. Results were typically positive for vimentin, α1-antitrypsin, and neuron-specific enolase. Progesterone receptors, but usually not estrogen receptors, were variably present. Synaptophysin, cytokeratin, and chromogranin A were expressed only focally in a few tumors. Ki-67 was detected in three patients with pancreatic parenchyma invasion. Figure 3 shows the histopathologic image results.

### Follow-up

Follow-up included clinical examination, routine laboratory tests, abdominal US, and CT or MRI every 3 months. The patients were followed up for a mean duration of 48.2 months, (range 2 to 90 months) and all 17 patients were alive with no evidence of disease recurrence or metastasis.

### Predictive factors of malignancy

On univariate analysis, none of the features, including age, tumor size, tumor location, increased tumor markers, and tumor characteristics, was predictive of malignant SPTs (Table 2).

**Table 2:** Predictive factors of malignant SPTs

**Clinicopathologic factors**	**Malignant (*n *= 4)**	**Benign (*n *= 13)**	***P *value**
**Mean age (years)**	34.4(19-55)	25.3(11-53)	0.11
**Symptoms**			
Present	3	11	0.65
Absent	1	2	
**Tumor location**			
Body or tail	4	6	0.16
Head	0	5	
Neck	0	2	
**Tumor size (cm)**			
< 5	1	5	0.55
> 5	3	7	
**Tumor markers**			
Increased	1	1	0.35
Normal	3	12	
**Calcification**			
Present	1	3	0.94
Absent	3	10	
**Hemorrhage or necrosis**			
**Present**	2	4	0.48
**Absent**	2	9	
**Tumor feature**			
Solid and cystic	1	9	0.11
Solid	3	4	

## Discussion

Solid pseudopapillary tumor of the pancreas is a rare neoplasm with a low malignant potential, usually affecting young women in the second or third decade of life. The pathogenesis of the tumor is unknown, although its tendency to affect young women has suggested that sex hormones may be involved in the origin of SPT. However, no differences in immunohistochemical stains for sex hormone-receptor proteins or in clinicopathologic characteristics had been found attributable to sex alone [5]. Sun *et al*. [6] reported that 62.5% of SPT patients had been infected by Hepatitis B virus (HBV), which may be involved in the pathogenesis of SPTs. However, this association has not been confirmed by other researchers.

The clinical presentation of SPTs is usually unspecific and two or more symptoms usually coexist. Most of the patients presented with unclear clinical features, including abdominal pain, abdominal discomfort, poor appetite, and nausea, which are related to tumor compression of the adjacent organs. Because patients lack distinctive symptoms, the majority of these tumors are diagnosed during complementary imaging investigations, such as CT or US of the abdomen. On US or CT, the lesion is usually seen to be large, and its internal structure ranges from cystic thick-walled or with an inner irregular margin to a predominantly solid mass with some cystic component [7]. On dynamic contrast-enhanced CT, the tumor is enhanced less than the adjacent normal pancreas [8]. Magnetic resonance imaging is better than CT in differentiating the cystic or solid component inside the tumor and providing information about resectability. The use of FNAC, either percutaneously or endoscopic ultrasound guided, can help distinguish SPTs from other pancreatic tumors. However, seeding of the needle tract by neoplastic cells and such complications as bleeding, pancreatic fistula, and biliary fistula during the procedure have also been reported [9]. Despite widespread availability of high-quality imaging systems, preoperative diagnosis was difficult. Only six patients were diagnosed as or suspected of SPTs in our series, and the misdiagnosis rate in other groups was reported as ranging from 38.5% to more than 70% [10,11]. According to our experience, data from CT or MRI scans combined with age and sex should be sufficient for the decision to operate, and FNAC should be performed where the radiological diagnosis is not clear enough.

Currently, complete aggressive surgical resection is the treatment of choice for SPTs, even in the case of local invasion or metastasis [12]. The surgical approach depends on the location, size, and nature of the neoplasms, as well as the time of surgery [10]. Intra-operative frozen section may be helpful to ascertain the adequate of the resection margins. Extensive lymphatic dissection is not warranted, as SPTs rarely have lymph node metastases. For the case of local invasion or metastases, there is also a consensus that surgical therapy should be performed [13]. Because of the excellent outcomes after complete resection [14], surgeons should always aim for complete *en-bloc* resection including adjacent structures preferably with microscopically clear margins. In our study, the infiltrated splenic vein and adjacent tissues were resected *en bloc* and a long-term survival was observed in these patients. Resection of distant metastases should be performed at the time of primary resection or even for recurrences. This aggressive approach is supported in some studies, which showed that most patients were alive at long-term follow-up after extended resection [12,15].

In our study, four patients with splenic vein infiltration or pancreatic parenchyma invasion were diagnosed as malignant SPTs. Some studies have shown a correlation between tumor size above 5 cm, tumor necrosis, the male sex, and SPTs with malignant potential [16,17]. However, several univariate analyses indicated that clinical factors, including sex, age, tumor size, tumor location, increased tumor markers, and tumor characteristics were not intensively related to the malignant potential of SPTs [4,10,18]. These results were consistent with that in our study. Moreover, we found that positive immunoreactivity for Ki-67 was detected in three patients with pancreatic parenchyma invasion. Our findings are similar to the report from Yang [10] and indicate that the detection of Ki-67 may correlate with the malignancy and poor outcome of SPTs. However, these results are only limited to a small sample of SPTs, and more cases should be detected for Ki-67 and other new biomarkers in further studies.

Solid pseudopapillary tumors are readily diagnosable, based on their pathological and immunohistochemical features. The tumors contain a mixture of solid, cystic, and pseudopapillary patterns in various proportions. The solid portions of the tumor are composed of uniform and polygonal epithelioid cells with well-vascularized stroma and a discohesive arrangement [14]. Immunohistochemically, SPTs are typically positive for vimentin, α1-antitrypsin, α1-antichymotrypsin, and neuron-specific enolase [19], but the unique immunohistochemical features with expression of CD56 and CD10 were not consistent in recent studies. Cells from SPTs may also reveal focal immunoreactivity for cytokeratin and synaptophysin, demonstrate abnormal nuclear localization of β-catenin and the presence of progesterone receptors and may express galectin-3, all of which are useful in differentiating SPTs from endocrine pancreatic tumors [20].

The prognosis of SPTs is good, even with local recurrence, as well as metastases or invasions. More than 95% of patients with SPTs limited to the pancreas are cured by complete surgical excision [20]. Local recurrence is reported to be less than 10%, and usually within 4 years of surgery [11]. Recurrence, local invasion, and limited metastases are not contraindications for resection, and long-term survival has also been observed in patients with malignant SPTs. The overall 5-year survival was estimated to be 95% in a review of 718 patients reported in the English literature [21]. Owing to the favorable prognosis and excellent long-term survival, even in the presence of local recurrence or stable metastases, predictive factors of survival are difficult to identify.

## Conclusions

Solid pseudopapillary tumors are infrequent, typically affecting young women without notable symptoms. Their behavior is relatively indolent and largely benign, however, surgical resection is warranted even in the presence of local invasion or metastases as patients demonstrate excellent long-term survival. Further studies should aim at acquiring more understanding of SPTs and establishing guidelines for SPT diagnosis and treatment.

## Consent

Written informed consent was obtained from the patient for publication of this report and any accompanying images.

## Abbreviations

CP: central pancreatectomy; CT: computed tomography; FNAC: fine needle aspiration cytology; H&E: hematoxylin and eosin; MRI: magnetic resonance imaging; SPT: solid pseudopapillary tumor; US: ultrasonography.

## Competing interests

The author(s) declare that they have no competing interests.

## Authors’ contributions

Wang X-G, Ni Q-F, Fei J-G, Zhong Z-X, Yu P-F designed and conducted the study, analyzed the data, and helped to write the manuscript. Yu P-F is the principal investigator, revised and edited the manuscript. All authors approved the final manuscript.
